# Exploring a New Pathophysiological Association in Acne Vulgaris and Metabolic Syndrome: The Role of Biogenic Amines and Glutathione Peroxidase

**DOI:** 10.3390/medicina60030513

**Published:** 2024-03-21

**Authors:** Alexa Florina Bungau, Delia Mirela Tit, Manuela Stoicescu, Lavinia-Cristina Moleriu, Mariana Muresan, Ada Radu, Mihaela Cristina Brisc, Timea Claudia Ghitea

**Affiliations:** 1 Doctoral School of Biomedical Sciences, Faculty of Medicine and Pharmacy, University of Oradea, 410087 Oradea, Romania; prada.alexaflorina@student.uoradea.ro (A.F.B.); mmuresan@uoradea.ro (M.M.); adaroman96@gmail.com (A.R.); 2 Department of Preclinical Disciplines, Faculty of Medicine and Pharmacy, University of Oradea, 410073 Oradea, Romania; 3 Department of Pharmacy, Faculty of Medicine and Pharmacy, University of Oradea, 410028 Oradea, Romania; timea.ghitea@csud.uoradea.ro; 4 Department of Medical Disciplines, Faculty of Medicine and Pharmacy, University of Oradea, 410073 Oradea, Romania; briscristina@yahoo.com; 5 Department III of Functional Sciences, “Victor Babes” University of Medicine and Pharmacy, 300041 Timisoara, Romania; moleriu.lavinia@umft.ro

**Keywords:** metabolic syndrome, acne severity, glutathione peroxidase, oxidative stress, biogenic amines

## Abstract

*Background and Objectives*: Metabolic disorders cause many skin issues, including acne vulgaris. This research investigated the function of glutathione peroxidase (GTPx) and biogenic amines as a potential novel pathophysiological link between metabolic syndrome (MetS) and acne vulgaris. *Materials and Methods:* The patients were distributed into two groups: metabolic precondition (MPG, *n* = 78) and control (CG, *n* = 81). To determine the extent of acne and metabolic preconditioning, patients were subjected to extensive clinical/paraclinical investigations. Additionally, catecholamine levels in urine and GTPx levels in blood were measured. *Results:* Mild acne was more common in the CG (32.1 vs. 6.4, *p* < 0.001), and severe acne was more common in the MPG (61.54 vs. 25.9, *p* < 0.001), with the average age being substantially higher in the MPG (23.81 vs. 21.05, *p* = 0.002). Significant variations were observed in the paraclinical levels for catecholamines (*p* < 0.05). In the MPG, most severe acne patients were overweight (52.1%), insulin-resistant (48.8%), or obese (47.9%). Moderate acne was most often linked to obesity (56%), overweight (44%), and insulin resistance (20%). Patients with severe acne (48.83%) had a considerably greater incidence of insulin resistance syndrome (*p* = 0.039) than those with moderate or severe acne (20%). The presence of two or three metabolic disorders considerably raised the risk of severe acne. Significant differences between groups were observed only in the subgroup of patients with severe acne, with lower values in the MPG (*p* = 0.015). Significant differences between groups were observed regarding the subgroup of patients with severe acne, with lower DTPx values in the MPG. At the group level, only CG patients with severe acne had reduced GTPx levels. Significant differences in catecholamine values were seen between groups (*p* < 0.05), independent of acne severity, except for adrenaline in mild acne patients (*p* = 0.059). *Conclusions:* The complex connection between GTPx and catecholamines in MetS suggests a significant role of these factors in the pathogenesis of acne associated with this condition, opening new perspectives in the research and treatment of acne in the context of MetS.

## 1. Introduction

A persistent global public health care system concern is the rising prevalence of metabolic syndrome (MetS), a complex condition characterized by several risk factors, such as insulin resistance, abdominal obesity, dyslipidemia, and hypertension [1]. The unusual (at first glance) connection between skin conditions and MetS has attracted the attention of academics in recent years. Many skin problems, including acne vulgaris, are linked to metabolic dysfunctions [2]. Multiple investigations have revealed that individuals with MetS have reduced levels of antioxidant enzymes in their plasma and elevated levels of markers indicating oxidative damage, particularly lipid peroxidation, in comparison to healthy individuals. This observation suggests that the presence of MetS may lead to the onset of an oxidative stress condition [3].

The pathophysiology of acne vulgaris is complex and includes multiple factors. New research links oxidative stress to the development of acne vulgaris, adding it to other pathogenetic markers like hyperkeratinization, increased sebum production, and immunoinflammatory responses [4]. Recent research has indicated antioxidants, either applied topically or taken orally, as a potential preventative treatment for skin photoaging and ultraviolet-induced malignancy [5]. Due to the connection between acne, the generation of ROS, and heightened inflammation, it is possible that the skin’s antioxidant defense system may be diminished [6]. The skin specimens taken from acne vulgaris lesions had a considerably reduced level of glutathione peroxidase (GPx) compared to those from normal skin. Furthermore, there was an inverse correlation between the severity of acne and the level of GPx [7]. In contrast, the concentration of malondialdehyde (MDA) exhibited a notable increase and showed a positive correlation with the severity of acne. Superoxide dismutase (SOD), catalase (CAT), and glutathione (GSH) were also reduced in the skin of people with acne [8].

In addition, acne patients are more likely to suffer from social anxiety, which is associated with elevated levels of oxidative stress. Some studies have found that people with acne have far reduced levels of antioxidant enzymes in their blood, such as GTPx and superoxide dismutase [9,10]. In MetS, mitochondrial dysfunction and the accumulation of reactive oxygen species significantly increase oxidative stress. This condition favors the development of acne by inducing inflammation, activating androgen receptors, and disrupting sebaceous cell homeostasis. GTPx, through its ability to reduce oxidative stress, thus becomes an important component in maintaining the integrity of the skin [11,12,13].

Catecholamines, such as adrenaline and noradrenaline, are involved in the stress response and impact the immune system and inflammation. In MetS, the dysregulation of these catecholamines can contribute to chronic inflammation, which favors acne onset. Also, catecholamines can stimulate the production of sebum and affect the function of the sebaceous glands, contributing to the formation of acne lesions [14,15,16,17]. Catecholamines are hormones produced primarily by the adrenal glands in response to physical or psychological stress. These chemicals play a crucial role in activating the sympathetic nervous system, preparing the body for fight or flight. In MetS, hyperactivity of the sympathetic nervous system can lead to the excessive release of catecholamines, creating an environment favorable to the development of chronic inflammation [17,18]. Adrenergic receptors are present on sebaceous glands, and their stimulation by catecholamines can have a significant impact on sebum secretion. An excess of sebum can create a favorable environment for bacteria multiplication and acne development. In addition, the stimulation of catecholamines may influence sebaceous cell differentiation and apoptosis, contributing to the specific pathology of acne [17]. Another aspect of MetS that becomes relevant is insulin resistance. Catecholamines may contribute to this resistance through various mechanisms, including the inhibition of insulin action [19].

From this perspective, GTPx and catecholamines seem to play a significant role in the pathogenesis of acne associated with metabolic syndrome. Nevertheless, few studies have looked at the relationship between oxidative stress and acne severity in people who have both metabolic syndrome and acne, and none of them provide a comprehensive catecholamine-level explanation for this relationship. The main objective of this research was to analyze the correlation between the levels of GTPx, catecholamines, and the severity of acne in patients with acne and metabolic preconditioning. The obtained results can open new research perspectives and can be a starting point to find new therapeutic approaches.

## 2. Materials and Methods

### 2.1. Patients and Methods

For this study, 159 patients diagnosed with acne between November 2022 and December 2023 in a private clinic (Pelican Hospital, Oradea, Romania) and a few private dermatology offices, in collaboration with a private dietetics office (Echolaboratoare, Oradea, Romania), were included in this cross-sectional analysis to determine the metabolic preconditioning impact on acne severity, GTPx, and biogenic amines (adrenaline, noradrenaline and dopamine).

Patients underwent comprehensive clinical and paraclinical evaluations to ascertain the severity of acne and the presence or absence of metabolic preconditioning, as well as to measure urinary levels of catecholamines and blood levels of GTPx.

The severity of the acne was evaluated by dermatologists using the global acne severity scale (GEA) [20], according to the criteria from one of our previous studies [21]. Individuals with a score of 1 or 2 were categorized as presenting “mild acne”. This type of acne is characterized by a small number of closed or open comedones, relatively few papules, and an easily identifiable afflicted area that does not exceed half of the face. “Moderate acne” was the classification given to patients with a score of 3. Over half of the face can be affected by this form of acne, which is marked by a multitude of open and closed comedones, pustules, and papules. Alternative situations include inflammatory and non-inflammatory lesions, a single nodule, or both. The patients were included in the “severe acne” category if their score was 4 or 5. This condition can spread over the whole face, causing a multitude of pustules and papules as well as unusual nodules and open or closed comedones. Additionally, these individuals exhibit severe inflammatory damage all over their faces, along with lesions and nodules.

The metabolic preconditioning was established by measuring blood pressure (HBP), lipid profile (low-density lipoprotein (LDL), high-density lipoprotein (HDL), and triglycerides (dyslipidemia)), fasting blood glucose, glycosylated hemoglobin (HbA1c), blood glucose collected at some point during the day (diabetes), insulin resistance syndrome (HOMA-IR), and body mass index (BMI), which represented the weight status [21].

Several analytical techniques were used to evaluate various parameters, including HDL and LDL cholesterol using direct colorimetric evaluation; cholesterol using oxidase-peroxidase; and triglycerides using enzymatic glycerol-3-phosphate oxidase. When the LDL cholesterol level rises above 160 mg/dL, the HDL cholesterol level falls below 40–50 mg/dL, or the triglyceride level rises above 150 mg/dL, these values indicate dyslipidemia [22]. The subjects had their baseline glucose levels measured using the hexokinase method. Glucose levels between 100 and 125 mg/dL were considered high, while those below 100 mg/dL were considered normal [23]. Blood insulin levels were measured with the use of a chemiluminescent enzyme immunoassay. The method presented in Table 1 was used to compute homeostasis of a minimum measurement of insulin resistance (HOMA-IR) from the basal level of glucose and the immunoreactive insulin value [24]. The concentration of glycosylated hemoglobin in blood samples taken from patients using the EDTA anticoagulant was determined using the high-performance liquid chromatography method in order to diagnose diabetes, with a value greater than 6.5% being set [22].

The most recent guidelines were followed for measuring blood pressure, and BMI was used to estimate weight status. BMI values between 25 and 29.9 kg/m^2^ were considered overweight, while a BMI greater than 30 kg/m^2^ indicated obesity [25]. Hypertension was characterized by a blood pressure greater than 130/85 mm Hg [26].

A metabolic preconditioning group (MPG, *n* = 78) was comprised of patients who had at least one metabolic disorder. The control group (CG, *n* = 81) consisted of patients who were diagnosed with acne but did not have any other associated condition. Patients with endocrine or other metabolic illnesses, as well as those taking oral contraceptives, isotretinoin, systemic antibiotics, or antiandrogens, were excluded from the study because they were thought to introduce bias.

To determine GTPx activity, a photometric method is employed, involving the analysis of whole blood using EDTA anticoagulant at a temperature of 2–8 °C and a minimum volume of 2 mL. Its normal values are in the range of 4171–10,881 U/L^4^.

Biogenic amine analysis encompassed the scrutiny of dopamine (reference range 125–250 µg/g creatinine), adrenaline (reference range 3–12 µg/g creatinine), noradrenaline (reference range 25–55 µg/g creatinine), and the noradrenaline-to-adrenaline ratio (reference range 3–7). These analyses took place in an analytical laboratory utilizing enzymatic, colorimetric, and spectrophotometric techniques, as well as immuno-enzymatic tests. To determine the presence of stress hormones in the body, specific tests utilizing urine samples were employed, conducted by CTL and Ortholabor GmbH in Bad Zwischenahn, Germany (CTL and Ortholabor GmbH, Bad Zwischenahn, Germany).

### 2.2. Statistical Design

To describe the studied and control group, a complex statistical analysis was conducted, starting with a descriptive analysis, by calculating the central tendency and dispersion parameters and developing frequency tables. For the data distribution, a Shapiro–Wilk test was used, from which we established that the data were not normally distributed. Thus, non-parametrical tests had to be applied as follows: the Mann–Whitney test for two different groups and the Kruskal–Wallis test for more than two different groups. To test the data association, a correlation model was used, and for data dependence, the Spearman parameter was calculated. For numerical variables, Pearson’s coefficient was applied to test the strength of the association between the tested variables. The level of significance was set at α=0.05 for the whole study. The data were gathered using the Microsoft Excel program, and the JASP v18.3 program was used for the statistical analysis.

## 3. Results

The relevant characteristics of the patients in the two groups are presented in Table 1. The data analysis indicates significant differences (*p* < 0.05) between the two groups at the level of some demographic parameters, as well as for clinical and paraclinical parameters. In the MPG, the average age was significantly higher than in the CG (23.81 vs. 21.05, *p* = 0.002), and severe acne had a significantly higher prevalence (61.54 vs. 25.9, *p* < 0.001), while mild acne was significantly more prevalent in the CG (32.1 vs. 6.4, *p* < 0.001). The average value of the body mass index was significantly higher in the MPG vs. the CG (32.4 vs. 24.72, *p* < 0.001).

At the paraclinical parameters level, significant differences were recorded in the case of catecholamines (*p* < 0.05).

**Table 1:** Relevant characteristics of the patients in the two groups.

Characteristics	CG (*n* = 81)		MPG (*n* = 78)		*p* ^1^
	No.	%	No.	%	
Gender					
Women	63	77.78	57	73.08	0.491
Men	18	22.22	21	26.92	
Environment					
Urban	70	86.4	57	73.1	0.036 *
Rural	11	13.6	21	26.9	
Age (years)					
14–25	77	4.9	52	33.3	<0.001 *
>25	4	95.1	26	66.7	
Average (mean ± SD)	21.05 ± 3.35		23.81 ± 4.84		0.002 ^2,^*
Acne stage					
Mild acne	26	32.1	5	6.41	<0.001 *
Moderate acne	34	42.0	25	32.05	
Severe acne	21	25.9	48	61.54	
Body mass index (kg/m^2^)	24.72	1.09	32.40	4.79	<0.001 ^2,^*
**Paraclinical parameters**	**Mean**	**SD**	**Mean**	**SD**	*p* **^2^**
Fasting blood glucose (mg/dL)	84.43	3.96	87.03	8.49	0.007 *
HOMA-IR (insulin (µU/mL) × glucose level (mg/dL))/405	1.22	0.63	2.55	2.00	<0.001 *
LDL cholesterol (mg/dL)	123.53	20.78	123.37	24.14	0.573
HDL cholesterol (mg/dL)	57.77	7.55	47.73	4.86	<0.001 *
Triglycerides (mg/dL)	133.96	9.58	139.03	14.12	0.669
Blood pressure (mm Hg)	107/68	0.3/0.04	110/72	0.6/0.23	0.239
Glutathione peroxidase (U/L^4^)	6015.33	1345.49	5680.37	1167.02	0.114
Dopamine (µg/g creatinine)	323.81	163.03	549.67	183.20	<0.001 *
Noradrenaline (µg/g creatinine)	38.12	13.39	61.55	12.82	
Adrenaline (µg/g creatinine)	4.79	3.78	11.42	1.88	
NADR/ADR	7.96	6.45	5.39	3.41	

^1^ Chi-square test, ^2^ Mann–Whitney test, * significant values, SD—Standard deviation.

Due to significant differences in age and environment between groups, an extensive statistical analysis was conducted to assess their potential influence on the studied parameters, regardless of metabolic preconditioning. Two distinct groups were formed based on the environment (EGs) (urban vs. rural), respectively, depending on age (AGs) (>25 vs. <25), and the Mann-Whitney test was applied. The results of the analysis indicated insignificant differences (*p* > 0.05) in both scenarios (Table 2). The age variable was analyzed both as a categorical variable (<25 years vs. >25 years) and as a numerical variable (mean ± SD); to complete this analysis, a correlation model was applied to test a possible association between age (as a numerical variable) and the numerical parameters evaluated in the study, finding no significant correlations (*p* > 0.05) (Table 3).

**Figure 1:**
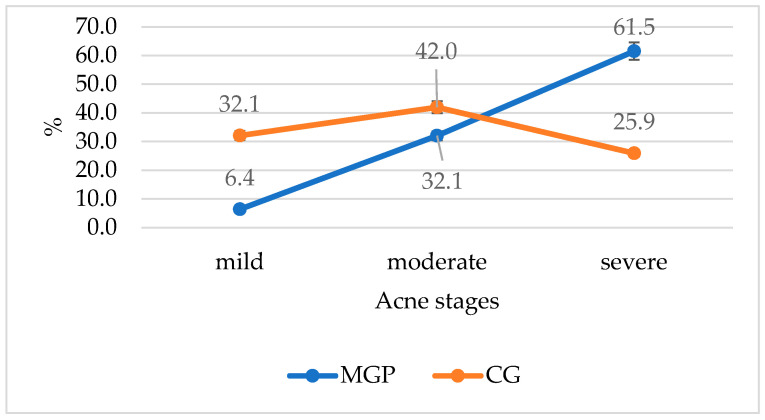
The frequency of acne stages in both groups. MGP—metabolic precondition group, CG—control group.

In the CG, most patients had moderate acne (42%), followed by mild acne (32.1%) and severe acne (25.9), without significant differences (*p* = 0.203). The most common form of acne in the MPG group was severe acne, which affected over 60% of patients (61.5%), significantly higher than moderate acne (32.1, *p* < 0.001) and mild acne (6.41%, *p* < 0.001). Figure 1 shows a line graph plotting the frequency of acne stages in both groups.

In the MPG, among the patients with severe acne, most subjects were overweight (52.1%), presented insulin resistance (48.8%), or were obese (47.9%). Dyslipidemia, diabetes, high glucose levels, and high blood pressure (HBP) were associated with severe acne in 12.5%, 4.2%, and 2.1% of patients. Moderate acne was most frequently associated with obesity (56%), followed by overweight (44%) and insulin resistance (20%). Patients with mild acne were all overweight (100%), and 20% presented insulin resistance. Insulin resistance syndrome was significantly more present (*p* = 0.039) in patients with severe acne (48.83%) compared to those with moderate or severe acne (20%) (Table 4).

**Figure 2:**
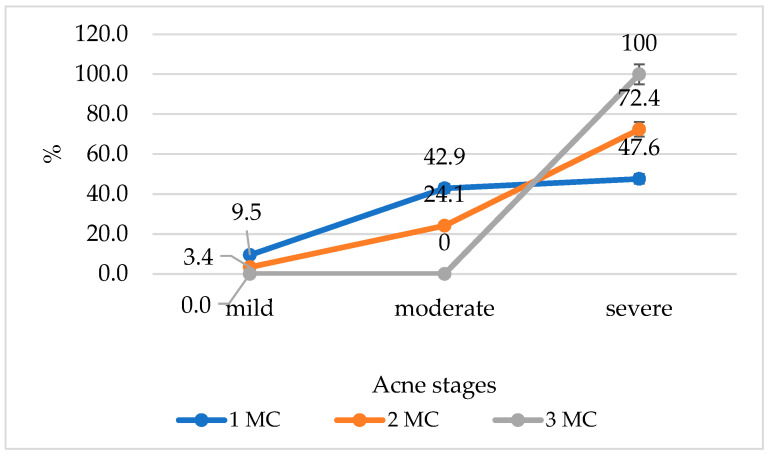
The acne severity based on the number of metabolic conditions. MC—the number of metabolic conditions.

Furthermore, 43.75% of severe acne patients had two overlapping metabolic diseases, and 14.6% had three overlapping metabolic diseases, whereas only 28% of moderate acne patients and 20% of mild acne patients had two overlapping metabolic diseases. Figure 2 shows a line graph plotting acne severity based on the number of metabolic conditions.

To investigate the relationship between acne severity and blood GTPx levels, or the level of catecholamines in urine, data were compared between groups (Mann-Whitney test) and within each group (Kruskal–Wallis test), depending on acne severity. Significant differences between groups were observed only in the subgroup of patients with severe acne, with lower DTPx values in the MPG (*p* = 0.015). At the group level, significant differences were recorded only in the CG, with patients with severe acne having a significantly lower level of GTPx. Catecholamine values differed significantly between groups (*p* < 0.05), regardless of the degree of acne, except from adrenaline in the subcategory of patients with mild acne (*p* = 0.059) (Table 5).

For determining the degree of association between the severity of acne and the studied variables, we applied a correlation model and calculated the Spearman coefficient since there are numerical and ordinal variables in the analysis, as presented in Table 6. For the studied group (MPG), a significant positive association was found for the adrenaline and noradrenaline variables $\left(\rho =0.326,\;p=0.004;\;\rho =0.365,\;p=0.001\right)$; significance was found when HOMA and fasting blood glucose were tested, also with a positive association $\left(\rho =0.344,\;p=0.002;\;\rho =0.275,\;p=0.015\right)$. For the control group (CG), a significant negative association was found in the case of GTPx $\left(\rho =-0.562,\;p<0.001\right)$, and there was a positive significant association found for noradrenaline variables $\left(\rho =0.244,\;p=0.028\right)$ and for HOMA, BMI and fasting blood glucose $\left(\rho =0.368,\;p<0.001;\;\rho =0.496,\;p<0.001;\;\rho =0.451,\;p<0.001\right)$.

## 4. Discussion

Acne vulgaris and metabolic disorders have been linked in a number of studies, indicating the role that different hormones and lipid imbalances play in the development and course of acne. Overall, the results of this research showed that there is a statistically significant correlation between the degree of severity of acne and metabolic preconditioning on the one hand, and/or the circulating levels of blood catecholamines on the other hand. As far as we are aware, this is the first study to assess the complex relationship between metabolic preconditioning and the severity of acne, as well as the enzymatic activity of GTPx and catecholamines.

Moreover, 159 patients diagnosed with acne, with an average age of 22.43 years, were included in this cross-sectional analysis. Among them, only 24.52% were men; the rest were women, with a relatively uniform distribution in the study groups (*p* > 0.05). Previous studies have indicated mixed results regarding the prevalence of acne depending on gender, with some indicating a higher prevalence in women [27,28] and others indicating a higher prevalence in men [29,30]. These findings could be a consequence of variations in the features of the nation under study or the sampled population. Moreover, the predominance of women in our study group may be related to age, with some studies indicating that, after the age of 20, women saw dermatologists nearly 2.5 times more frequently for acne-related issues than men [31]. The fact that women with acne vulgaris have significantly greater levels of anxiety and sadness and a lower quality of life than men may also have an impact on this feature [32,33].

Age and the environment were variables with significant differences between groups, but the statistical analysis performed did not indicate a significant influence of them on the analyzed parameters. However, even if there were significant age differences between the groups, the mean age was relatively close (21.05 years in the CG vs. 23.81 years in the MPG). Early adulthood can still be characterized by metabolic changes caused by the transition from youth to adulthood. These changes may include changes to one’s body composition, hormone levels, or way of life [27,28]. However, the effect of age itself on the metabolic parameters assessed in this study may be very small during this period compared to younger ages or older adulthood. This aspect was confirmed by the results of the statistical analysis.

One of the less studied aspects of MetS is its connection to acne, a common skin condition. The pathophysiological processes associated with MetS and acne vulgaris include chronic inflammation and elevated levels of oxidative stress indicators, which are believed to mediate the association between the two diseases, according to recent studies [13]. Studies have shown that people with MetS often have reduced levels of GTPx compared to healthy individuals [29]. This deficit may contribute to antioxidant instability and increase cellular vulnerability to oxidative insults. The results of this study demonstrated that patients with severe acne had a significantly higher prevalence in the MGP. When comparing the two studied groups related to the stage of acne, the results showed also statistically significant differences (*p* < 0.001).

Recent studies show that the skin can immediately detect stress and is also a target of the body’s stress reactions. The skin, being the major organ in the body, serves as a barrier between the inside of the body and the outside world, acting as an immune system and regulating temperature and humidity [17].

Comparing the circulating catecholamines, our outcomes demonstrated significant differences between groups (MGP and CG) for dopamine, noradrenaline, adrenaline, and NADR/ADR, *p* < 0.001. Insulin and noradrenaline work separately to cause MetS. However, patients with MetS had higher urinary noradrenaline levels than those without this condition [31].

This study also explored the relationship between the severity of acne and the level of GTPx, respectively the level of catecholamines in patients with acne and metabolic preconditioning. The results indicated a significantly higher prevalence of severe acne in the study group patients and lower GTPx activity in this group but without significant differences. When the differences were analyzed according to the severity of acne, it was found that GTPx had significantly lower values in patients with severe acne and metabolic preconditioning compared to those with severe acne in the control group. In the MPG group, GTPx activity decreased with the increase in the degree of acne, without significant differences, while in the control group, patients with severe acne had significantly lower GTPx than those with moderate acne or severe acne.

Taking into consideration the stage of acne, the results of the Mann-Whitney test when comparing the two groups, and those of the Kruskal–Wallis test when comparing the variables in each group, we found a statistically significant difference for dopamine for both tests. These outcomes of the complex investigation of the relationship between the severity of acne and the level of catecholamines indicated that severe acne is associated with a significant increase in the level of dopamine, regardless of the group. A significantly higher level of dopamine was also found in the group with metabolic preconditioning compared to the control group regardless of the severity of the acne. Dopamine is one of the most important messenger substances in the brain, and its action is mainly of a stimulating nature. In the case of acute stress, the secretion of dopamine can increase. An elevated dopamine level can reduce the ability to re-coup and regenerate, causing fatigue throughout the day and difficulty concentrating [34,35,36,37].

For noradrenaline, the relevant statistics reached between groups and into the MPG, and for adrenaline, we obtained a *p*-value of 0.001 between groups but only for patients with moderate and severe acne stage in the MPG. Noradrenaline and adrenaline had also significantly higher values in the MPG group compared to the CG, while the NADR/ADR ratio was significantly lower, regardless of the severity of the acne. For NADR/ADR, statistically significant differences were reached between groups for patients with mild and moderate acne and in the MPG. The increase in the level of adrenaline and noradrenaline and the decrease in the NADR/ADR ratio were significantly associated with the increase in the severity of acne only in the group with metabolic preconditioning. The noradrenaline/adrenaline ratio serves as a valuable indicator of overall stress level. A ratio of less than 3 usually indicates significant chronic stress.

Controlling catecholamine levels may represent important therapeutic directions in the management of MetS-associated acne. Strategies aimed at reducing stress, normalizing the activity of the sympathetic nervous system, or regulating the release of catecholamines may have a positive impact on the prevention and treatment of skin lesions in the context of MetS [38]. By exploring in detail the complex relationship between catecholamines and oxidative stress in MetS, more precise and personalized therapeutic strategies for affected patients can be developed, thereby contributing to the effective management of acne associated with this complex condition.

Using the Spearman coefficient, this study also assessed the degree of association between the severity of acne and the studied variables. The results showed a negative direct association, *p* < 0.001, between the GTPx level and the acne stage. That means that when the level of GTPx is low, the acne stage is more severe.

Studies have shown that GTPx can influence catecholamine metabolism, regulating the expression of adrenergic receptors [39,40,41]. Considering this complex interaction, therapeutic approaches should aim to improve GTPx activity [42,43]. Further research in this direction could reveal new target molecules and ways to regulate GTPx for the effective management of acne in MetS.

A positive direct association, with statistical significance, was also found between the circulating level of noradrenaline and acne stage, *p* < 0.001. When the level of noradrenaline is high in circulating blood, there is a greater risk of developing severe acne.

Through the detailed analysis of the interaction between GTPx and catecholamines in MetS, the mechanisms of acne pathogenesis can be better understood, and personalized therapeutic strategies can be developed for patients affected by this complex condition. Given these findings, the development of therapeutic strategies aimed at improving glutathione peroxidase activity and regulating catecholamine levels could provide new insights into the treatment of acne associated with MetS. These strategies could include dietary interventions, antioxidant supplementation, and therapies aimed at normalizing stress levels.

The additional measures can complement efforts to regulate stress and oxidative stress, thereby contributing to improved skin quality and the overall health of patients.

## 5. Conclusions

The results of this study showed that the complex relationship between metabolic preconditioning, circulating levels of GTPx, and catecholamines could represent a potential factor in the appearance and the development in severity of acne. In this context, the management of the acne treatment should take into account targeting the parameters mentioned above as an instrument for the improvement of therapy. However, further long-term research (involving a larger sample size; groups being more homogeneous in terms of age, environment, and gender; and following participants over time) is needed to produce more precise findings and to provide a deeper understanding of this intricate link/connection.

## Figures and Tables

**Table 2:** The differences for the tested variables depending on environment and age.

Parameters	EGs	*n*	Mean	SD	*p* ^2^	AGs		Mean	SD	*p* ^2^
BMI	U	127	28.543	5.177	0.726	>25	30	28.907	4.738	0.329
	R	32	28.577	5.165		<25	129	28.213	5.266	
Fasting blood glucose (mg/dL)	U	127	85.819	6.807	0.453	>25	30	85.677	4.367	0.500
	R	32	85.641	6.278		<25	129	85.783	7.133	
HOMA-IR ^1^	U	127	1.783	1.573	0.185	>25	30	1.947	1.469	0.443
	R	32	1.987	1.735		<25	129	1.823	1.644	
LDL cholesterol (mg/dL)	U	127	123.866	23.286	0.753	>25	30	123.867	20.257	0.654
	R	32	123.034	18.798		<25	129	123.033	22.960	
HDL cholesterol (mg/dL)	U	127	51.688	11.894	0.474	>25	30	50.892	11.521	0.470
	R	32	53.812	11.931		<25	129	54.608	11.857	
Triglycerides (mg/dL)	U	127	138.228	12.231	0.471	>25	30	138.085	12.894	0.224
	R	32	134.732	10.010		<25	129	134.905	8.794	
Glutathione peroxidase (U/L^4^)	U	127	5753.472	1205.422	0.054	>25	30	5697.809	1218.556	0.233
	R	32	5942.228	1448.781		<25	129	5997.891	1301.329	
Dopamine (µg/g creatinine)	U	127	434.355	205.990	0.811	>25	30	440.000	226.457	0.841
	R	32	439.125	211.776		<25	129	433.480	202.509	
Noradrenaline (µg/g creatinine)	U	127	50.014	18.236	0.498	>25	30	51.05	24.308	0.416
	R	32	49.656	14.910		<25	129	48.620	16.052	
Adrenalin (µg/g creatinine)	U	127	7.847	4.568	0.638	>25	30	8.110	2.319	0.322
	R	32	8.363	4.035		<25	129	8.100	4.418	
NADR/ADR	U	127	6.373	3.984	0.663	>25	30	6.295	3.241	0.056
	R	32	5.938	3.024		<25	129	6.002	4.328	

^1^ (insulin (µU/mL) × glucose level (mg/dL))/405, ^2^ Mann–Whitney test, EGs—environmental groups, AGs—age groups, SD—Standard deviation, BMI—body mass index, NADR/ADR—noradrenaline/adrenaline ratio, U—urban, R—rural.

**Table 3:** The degree of association between the age and the studied variables.

Correlation of Age with the Studied Variables	Pearson’s r Coefficient	*p*
BMI	−0.014	0.864
Fasting blood glucose	0.022	0.788
HOMA-IR	−0.105	0.187
LDL cholesterol	0.056	0.484
HDL cholesterol	−0.068	0.392
Triglycerides	0.159	0.055
Blood pressure	0.019	0.866
Glutathione peroxidase	−0.012	0.882
Dopamine	0.051	0.521
Noradrenaline	−0.011	0.889
Adrenaline	0.044	0.583
NADR/ADR	−0.166	0.056

BMI—body mass index, NADR/ADR—noradrenaline/adrenaline ratio.

**Table 4:** Patients’ distribution according to acne stage and the presence of each metabolic condition.

Patients MPG (*n* = 78)	Mild (*n* = 5)		Moderate (*n* = 25)		Severe (*n* = 48)		*p* ^1^
	*n*	%	*n*	%	*n*	%	
High glucose	0	0	1	4	2	4.2	0.898
Insulin resistance syndrome	1	20	5	20	22	48.83	0.039 *
Diabetes	0	0	0	0	2	4.2	0.527
Dyslipidemia	0	0	2	8	6	12.5	0.615
High Blood Pressure	0	0	0	0	1	2.1	0.729
Overweight	5	100	11	44	25	52.1	0.072
Obesity	0	0	14	56	23	47.92	0.079

^1^ Chi-square test, * significant values.

**Table 5:** The differences for the tested variables depending on acne stage.

Characteristics	CG (*n* = 81)		MPG (*n* = 78)		*p* ^1^
Glutathione peroxidase (U/L^4^)					
Mild acne	5548.79	1688.27	4855.38	118.36	0.081
Moderate acne	6089.29	760.39	5874.26	1348.97	0.154
Severe acne	6855.50	1085.19	5583.60	620.32	0.015
*p* ^2^	*p* = 0.001		*p* = 0.675		-
Catecholamines (µg/g creatinine)					
Dopamine					
Mild acne	250.10	162.89	545.19	2.68	0.001
Moderate acne	328.96	163.95	535.71	178.63	0.001
Severe acne	365.41	141.81	673.80	194.01	0.001
	*p* = 0.003		*p* = 0.005		
Noradrenaline					
Mild acne	33.05	13.12	52.60	0.55	0.007
Moderate acne	38.53	14.42	55.94	13.14	0.001
Severe acne	41.69	10.70	66.76	10.99	0.001
*p* ^2^	*p* = 0.098		*p* = 0.001		
Adrenaline					
Mild acne	3.34	4.49	11.00	0.00	0.059
Moderate acne	5.36	3.93	10.14	2.16	0.001
Severe acne	5.22	1.85	12.41	.98	0.001
*p* ^2^	*p* = 0.129		*p* < 0.001		
NADR/ADR					
Mild acne	9.70	5.60	4.77	0.05	0.001
Moderate acne	7.18	6.06	5.81	1.80	0.001
Severe acne	7.98	7.82	5.5	4.26	0.015
*p* ^2^	*p* = 0.064		*p* = 0.019		

^1^ Mann–Whitney, ^2^ Kruskal–Wallis Test, NADR/ADR—noradrenaline/adrenaline ratio.

**Table 6:** The degree of association between the severity of acne and the studied variables.

Studied Variables	MPG		CG	
	Spearman Coefficient $\left(\rho *\right)$	*p* *	Spearman Coefficient $\left(\rho *\right)$	*p* *
Glutathione peroxidase	−0.186	0.103	−0.562 *	<0.001 *
Adrenaline	0.326 *	0.004 *	0.180	0.108
Noradrenaline	0.365 *	0.001 *	0.244 *	0.028 *
NADR/ADR	−0.101	0.378	−0.062	0.583
Dopamine	−0.159	0.165	−0.169	0.132
HOMA index	0.344 *	0.002 *	0.368 *	<0.001 *
Body Mass Index	0.019	0.866	0.496 *	<0.001 *
LDL values	0.091	0.426	0.004	0.970
Fasting blood glucose	0.275 *	0.015 *	0.451 *	<0.001 *
HDL values	−0.020	0.863	0.171	0.127
TG values	0.051	0.655	0.212	0.057

* Significant values.

## Data Availability

Patients’ information is private and is archived in the electronic databases of the medical units where the research was done.
